# Pupillary Abnormalities with Varying Severity of Diabetic Retinopathy

**DOI:** 10.1038/s41598-018-24015-9

**Published:** 2018-04-04

**Authors:** Mukesh Jain, Sandeep Devan, Durgasri Jaisankar, Gayathri Swaminathan, Shahina Pardhan, Rajiv Raman

**Affiliations:** 1 Shri Bhagwan Mahavir Vitreoretinal services, 18, College Road, Sankara Nethralaya, Chennai, 600 006 Tamil Nadu India; 2 Elite School of Optometry, 8 GST Road, Chennai, 600016 Tamil Nadu India; 3 Vision and Eye Research Unit (VERU), Postgraduate Medical Institute, Anglia Ruskin University, Cambridge, CB1 1PT UK

## Abstract

Our aim is to study the dynamics of pupillary abnormalities in varying severity of diabetic retinopathy. A non-interventional case-control study with 405 eyes of 244 subjects with diabetes, and 41 eyes of 26 subjects with no history of diabetes was done. Diabetes group was classified according to retinopathy severity: no retinopathy, mild non-proliferative diabetic retinopathy (NPDR), moderate NPDR, severe NPDR and proliferative diabetic retinopathy (PDR). After dark adaptation, pupil size and flashlight response were captured with an infrared camera. Baseline Pupil Diameter (BPD), Amplitude of Pupillary Constriction (APC), Velocity of Pupillary Constriction (VPC) and Velocity of Pupillary Dilatation (VPD). Compared to controls, mean BPD decreased with increasing severity of diabetic retinopathy. Mean APC in control group was 1.73 ± 0.37 mm and reduced in mild NPDR (1.57 ± 0.39, p = 1.000), moderate NPDR (1.51 ± 0.44, p = 0.152) and found to be significant reduced in severe NPDR (1.43 ± 0.48, p = 0.001) and PDR (1.29 ± 0.43, p = 0.008). Compared to controls, mean VPC decreased progressively with increasing severity of retinopathy, with a maximal difference in the PDR group. Mean VPD as compared to the control group was significantly reduced in the no DR (p = 0.03), mild NPDR (p = 0.038), moderate NPDR (p = 0.05), PDR group (p = 0.02). We found pupillary dynamics are abnormal in early stages of diabetic retinopathy and progress with increasing retinopathy severity.

## Introduction

Diabetic Autonomic Neuropathy (DAN), although not very well explored or examined, is a common complication of diabetes. It affects the cardiovascular, gastrointestinal, genitourinary, sudomotor and ocular systems, and leads to considerable morbidity and mortality in subjects with diabetes^1^. Identification of these high-risk subjects is therefore crucial to improve the long-term outcomes of people suffering with diabetes.

Conventional diagnostic modality for assessing DAN include a battery of tests needing sophisticated and specific equipment, trained technicians and are time-consuming^2^. Moreover, these tests require active patient participation and compliance which limits its use in common clinical practice.

Dynamic pupillometry is an alternative technique that is simple, non-invasive, inexpensive and fast requiring minimal patient participation, and has been studied in various disease conditions affecting the Autonomic Nervous System (ANS), particularly diabetes^3–5^.

The Pupil Light Reflex (PLR) is under the direct control of the Autonomic Nervous System (ANS). The pupil’s size is controlled by circular (sphincter) and radial muscles of the iris. The circular muscle is innervated by the parasympathetic nervous system (PNS) while the radial muscle is innervated by the sympathetic nervous system (SNS) fibers. Thus, the PLR allows the evaluation of both the PNS and SNS dysfunctions. Dysfunction of the PNS causes relative mydriasis in light conditions and diminished constriction to light. Dysfunction of the SNS causes relative miosis of the pupil in the dark and increased re-dilatation lag.

Although, there are many studies that showed abnormal pupillary dynamics in diabetes, suggestive of autonomic dysfunction, its relation to the varying severity of diabetic retinopathy (DR) has not been explored extensively^4,6–10^. Clark studied ocular autonomic dysfunction in 28 diabetic subjects with proliferative diabetic retinopathy^11^. In a small cohort of 30 diabetic subjects, Datta *et al*. studied ocular autonomic dysfunctions in three broad groups of diabetic retinopathy: no retinopathy (n = 10), non-proliferative diabetic retinopathy (n = 10) and proliferative diabetic retinopathy (n = 10)^12^. In this study, we sought to examine the pupillary dynamics along the entire spectrum of DR: no DR, mild NPDR, moderate NPDR, severe NPDR and PDR, and compare it age matched normals.

## Study Population and Methodology

The Institutional Review Board of Vision Research Foundation approved this prospective study. 405 eyes of 244 subjects with diabetes above the age of 35 years were examined in patients attending a diabetic screening camp. 26 age-matched subjects with no history of diabetes were recruited as the control group. 41 eyes of these healthy subjects were analyzed.

Subjects who had an irregular and non-symmetrical pupil, iris abnormality including rubeosis iridis, optic nerve diseases, and on medication (local and systemic) known to affect the ANS were excluded from the study. We also excluded patients who had asymmetric DR grading between eyes. Systemic conditions known to affect the ANS like Parkinson disorder were also excluded. Informed consent was taken from all participants, and the tenets of the Declaration of Helsinki was followed.

After a brief initial history, preliminary ophthalmic examination on slit-lamp was done. Subjects were requested to sit for 15 minutes in a dark room, to allow for dark adaptation, before pupillography was carried out. Pupil measurements were performed with an 1/3 inch infrared camera and flash light (10 ws xenon flash lamp, Orion, Nidek Technologies, Italy). Pupil diameter was continuously measured with infrared light with wavelength ranging between 760 nm to 870 nm. Pupil size and the pupillary response to flashlight were captured and plotted against time.

**Figure 1:**
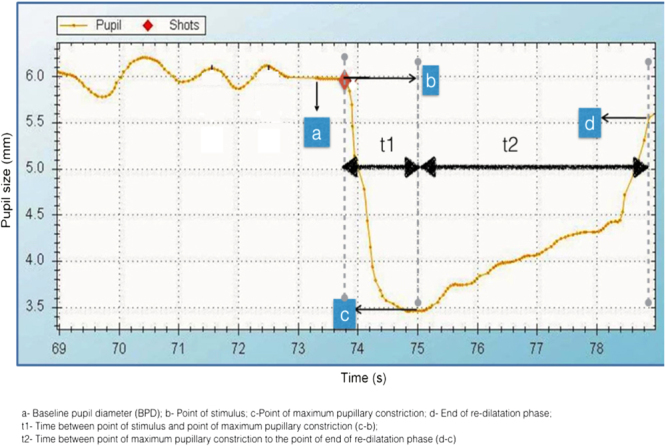
A typical pupillary light reaction graph obtained from the infrared pupillometer.

Following parameters were measured from the graph (Fig. 1): Dark-adapted Baseline pupillary diameter (BPD) was measured before exposure to the flash of light (point a). The amplitude of pupillary constriction (APC) to the flash of the light (in mm) and the time (t1) taken was measured from the graph (between point b and c). The velocity of pupillary constriction (VPC) was calculated as the speed of constriction (distance divided by the time) in mm/sec. The amplitude of pupil re-dilatation after maximum constriction (in mm) and time (t2) taken (in sec.) was measured from the graph (between point c and d). Velocity of pupillary dilatation (VPD) was calculated as the speed of re-dilatation (distance divided by time) in mm/sec.

Diabetic retinopathy was graded clinically using Klein’s classification (Modified Early Treatment Diabetic Retinopathy Study ETDRS classification). Retinal photographs were obtained after pupillary dilatation (Carl Zeiss fundus camera; Visucamlite, Jena, Germany); all patients underwent 45°, 4-field stereoscopic digital photography (posterior pole, nasal, superior, and inferior). All photographs were graded by two independent observers in a masked fashion; the grading agreement was high (κ = 0.83). Subsequently, the eyes were classified according to the following grouping: 147 diabetic eyes with no DR (no-DR), 53 eyes with mild non-proliferative diabetic retinopathy (NPDR), 146 eyes with moderate NPDR, 33 eyes with severe NPDR and 26 eyes with PDR.

A statistical package for Social Sciences-SPSS (version 21.0) was used for statistical analysis. All the data were expressed as mean ± SD. The age and baseline pupillary diameter were compared between groups using One-way ANOVA. Comparison between control group and different stages of DR group were made using General Linear Model Repeated Measures analysis to overcome the influence of inter-eye dependency since both the eyes were used for analyses in most of the subjects. The statistical significance was assumed at p values < 0.05.

### Synopsis

Dynamics of pupillary reaction in varying diabetic retinopathy severity was studied to understand autonomic nervous system dysfunction in diabeties. We found that alteration of pupillary dynamics even in early stages of diabetic retinopathy which progresses with increase in severity.

### Data availability statement

The datasets generated during and/or analysed during the current study are available from the corresponding author on reasonable request.

## Results

The diabetes group constituted 105 males and 139 females, with a mean age of 44 ± 8.5 years. The non-diabetes group constituted 15 males and 11 females, with a mean age of 43.1 ± 5.7 years. The mean age was compared between groups and found to have no statistically significant difference between group (p = 0.723).

**Figure 2:**
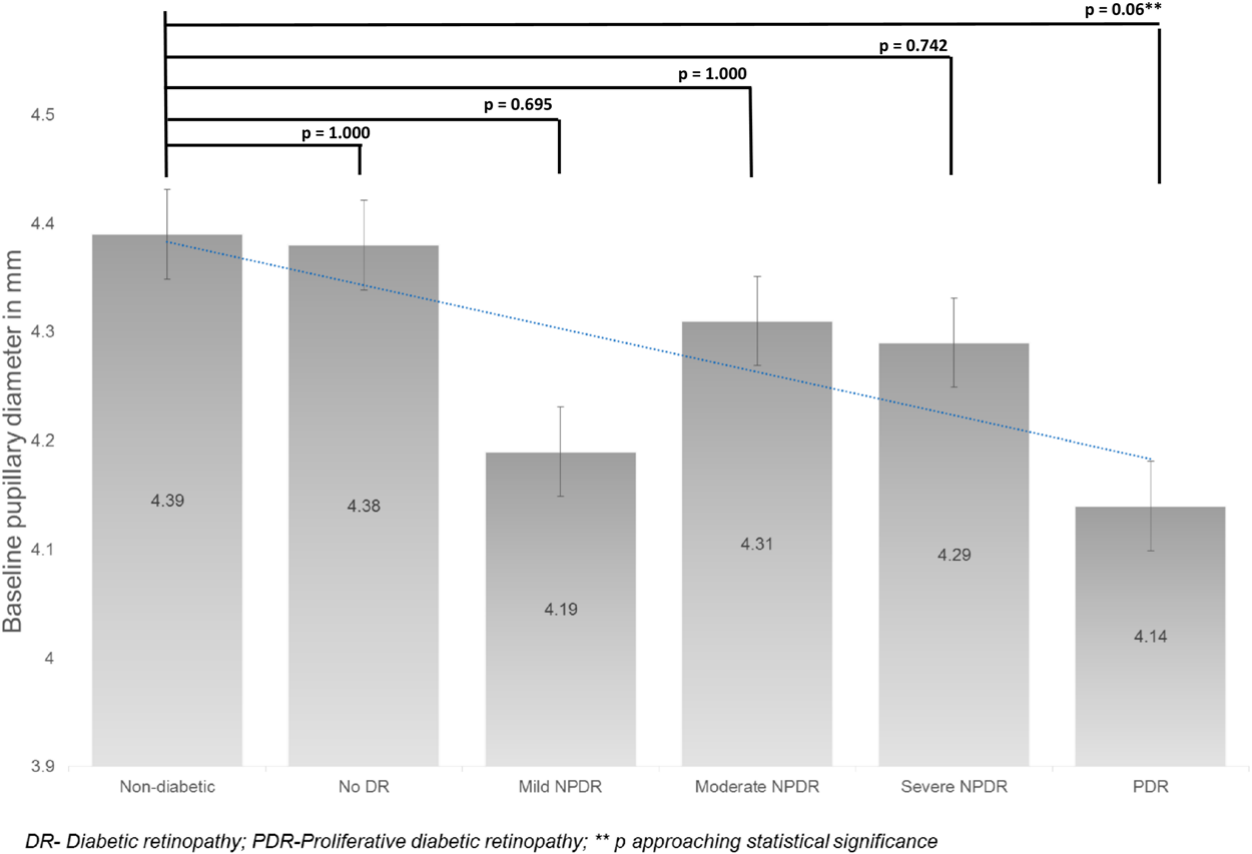
The mean baseline pupillary diameter (in mm) in various groups.

Table 1 shows the pupillary dynamics parameters expressed as mean and SD in the various groups. The mean BPD in the control group was 4.39 ± 0.60 mm. Compared to the control group, the mean BPD decreased with increasing severity of diabetic retinopathy, with a demonstrable trend towards significance (p = 0.06 in PDR group; Fig. 2). However, overall there was no statistically significant difference in BPD (p = 0.582).

**Table 1:** Pupillary dynamic parameters in various stages of DR.

***Parameters***	***No Diabetes***	***Diabetes***				
		***No DR***	***Mild DR***	***Moderate DR***	***Severe DR***	***PDR***
BPD	4.39 ± 0.60	4.38 ± 0.63	4.19 ± 0.50	4.31 ± 0.62	4.29 ± 0.75	4.14 ± 0.42**
APC	1.73 ± 0.37	1.69 ± 0.49	1.57 ± 0.39	1.51 ± 0.44	1.43 ± 0.48*	1.29 ± 0.43*
VPC	2.12 ± 0.38	2.12 ± 0.57	1.98 ± 0.54	1.96 ± 0.53	1.88 ± 0.59	1.86 ± 0.66
VPD	0.45 + 0.20	0.40 ± 0.16*	0.38 ± 0.18*	0.43 ± 0.22*	0.49 ± 0.30	0.38 ± 0.13*

APC: Amplitude of pupillary constriction; VCP: Velocity of pupillary constriction; VPD: Velocity of pupillary re-dilatation; **p approaching statistical significance; *p is statistically significant.

**Figure 3:**
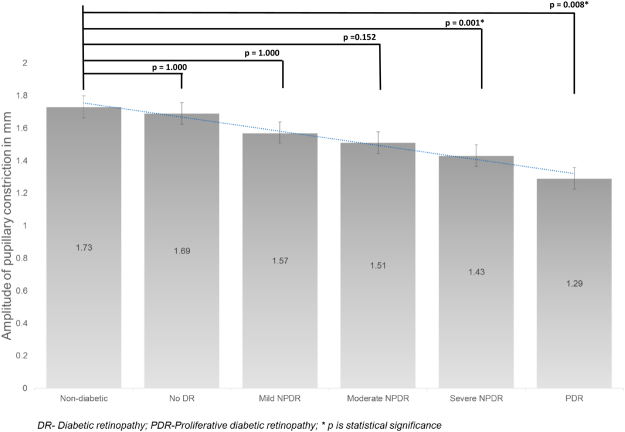
The mean amplitude of pupillary constriction (in mm) in various groups.

**Figure 4:**
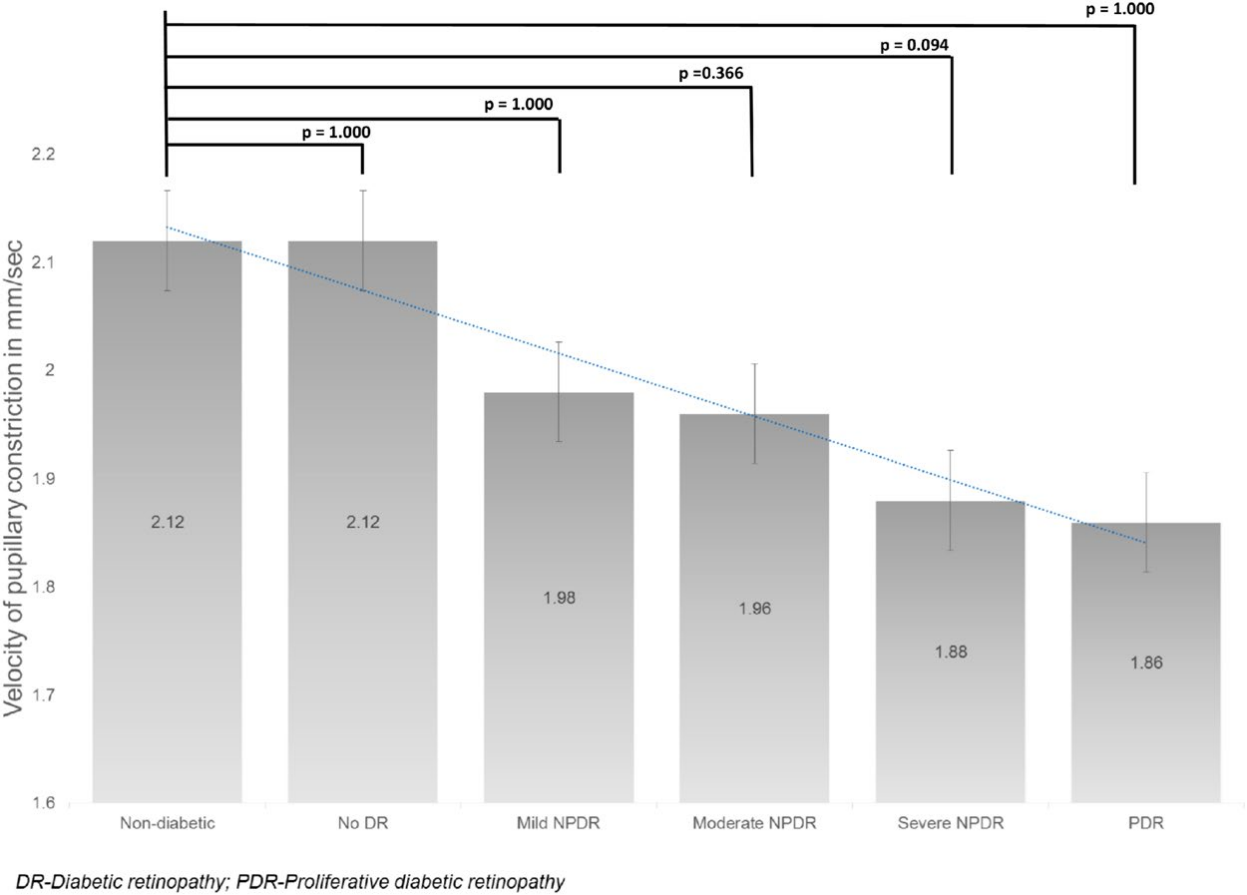
The mean velocity of pupillary constriction (in mm/s) in various groups.

**Figure 5:**
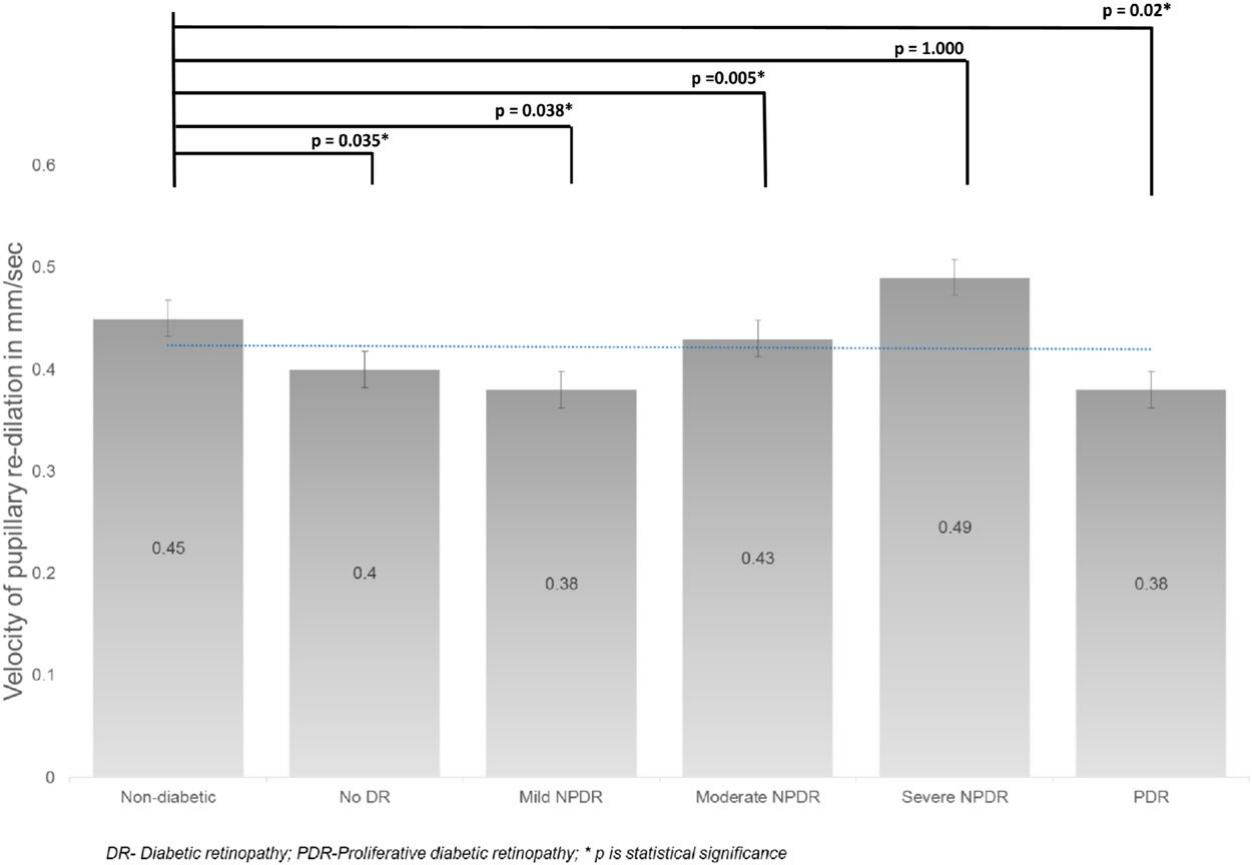
The mean velocity of pupillary re-dilation (in mm/s) in various groups.

The mean APC in the control group was 1.73 ± 0.37 mm (Fig. 3). Compared to the control group the mean APC decreased, however not statistically significant in no DR (1.69 ± 0.49, p = 1.000), mild NPDR (1.57 ± 0.39, p = 1.000) and moderate NPDR (1.51 ± 0.44, p = 1.000), and found to be statistically significant in severe NPDR (1.43 ± 0.48, p = 0.001) and PDR (1.29 ± 0.43 mm, p = 0.008). The mean VPC in the control group was 2.12 ± 0.38 mm/sec (Fig. 4). The mean VPC decreased with increasing severity of diabetic retinopathy, although not statistically significant. The mean VPD in the control group was 0.45 ± 0.20 mm/sec (Fig. 5). Compared to controls, the mean VPD decreased progressively in no DR (0.40 ± 0.16 mm/sec; p = 0.035), mild NPDR (0.38 ± 0.18 mm/sec; p = 0.038), moderate NPDR (0.43 ± 0.22 mm/sec; p = 0.005) and PDR (0.38 ± 0.13 mm/sec; p = 0.02).

## Discussion

This study found a smaller BPD, reduced APC, VPC and VPD in response to light in subjects with diabetes. Our results are in agreement with the previous studies^4,6–10^. More importantly, we found these abnormalities are present in subjects with early stages of diabetic retinopathy which increases progressively along the spectrum of retinopathy.

Smith and Dewhirst^10^, concluded that the resting pupillary diameter in the dark-adapted state is mainly under the SNS, and that smaller pupils in subjects with diabetes is a sign of sympathetic dysfunction. We found the mean BPD was smaller in patients with diabetes as compared to the non-diabetes group, although not significant (Fig. 2). However, it was interesting to note that in PDR group, this changes was approaching statistical significance, with the value of p = 0.058.

Although PLR as a whole is governed both by SNS and PNS, the different phases have differential innervation by SNS and PNS. During the constriction phase, PNS plays a dominant role, while contribution by the SNS is negligible. However, at the beginning of the re-dilatation phase, both PNS and SNS innervate the pupil^8^. Thus, the parameters relating to pupillary constriction (amplitude and velocity) may be considered as a PNS-related PLR parameters, uncontaminated by the SNS activity. Although it been argued that these parameters depend on the baseline pupillary diameter (influenced by the SNS), previous research suggests that PNS dysfunction is solely responsible for the constriction phase^5^. In the first meta-analysis to evaluate the sensitivity of PLR to diagnose parasympathetic dysfunction, Wang *et al*. found APC and VPC parameters to be highly sensitive^13^. We found that both APC and VPC decrease with increasing severity of DR (Figs 3 and 4). This observation of early PNS dysfunction which further increases along the spectrum of DR implies early parasympathetic neuronal damage in patients with diabetes, even in those who do not have DR.

We found the mean VPD in the diabetic group was significantly slower than the control group (Fig. 5). Because of the dual innervation (both PNS and SNS) during the re-dilatation phase of PLR, our finding relating to this simply denotes ANS dysfunction in diabetes. Any further interpretation of this finding is difficult.

We showed evidence of early ANS dysfunction in patients with diabetes, which increases progressively with the severity of retinopathy. Datta *et al*. showed evidence of significant PNS dysfunction in subjects with diabetes with NPDR as compared to healthy subjects^12^. Clark found abnormal pupil reflexes in 88.5% of patients with proliferative diabetic retinopathy^11^.

Evidence of significant impairment of PLR in patients with diabetes with severe grades of retinopathy, cannot be attributed exclusively to retinopathy severity. Perhaps it represents a significant correlation between diabetic complications, retinopathy and autonomic dysfunction. Smith suggested that ocular autonomic nervous system dysfunction occurs before cardiovascular autonomic dysfunction^14^. A strong association have also been shown between the severity of diabetic retinopathy and cardiovascular autonomic dysfunction in patients with diabetes^15^. Several pathways have been suggested in the pathogenesis of DAN. Hyperglycemia-induced activation of polyol pathway, Protein kinase C, increased oxidative stress, reduced nitric oxide production, autoimmune mechanisms, deficiency of neurotrophic growth factors, and formation of advanced glycosylation end products have been implicated^16–21^. These multifactorial pathways act through activation of polyADP ribosylation and depletion of ATP, resulting in cell necrosis and activation of genes involved in neuronal damage^22^.

Although ANS dysfunction is crucial for abnormal PLR, an additional contributory mechanism also results from structural changes of the iris in diabetes. Fujii *et al*. studied the ultrastructure abnormalities in iris specimen obtained from patients with diabetes mellitus^23^. They found significant structural changes in the dilator pupillae and constrictor pupillae muscles, their nerves endings and evidence of nerve fibers loss. In an interesting study done by Nitoda *et al*., they showed that nerve fiber alterations of the corneal sub-basal nerve plexus progress in parallel with DR and peripheral diabetic neuropathy using confocal microscopy^24^.

The major strengths of the study are substantial sample size and standard photographic diagnosis of diabetic retinopathy by two independent graders in a masked fashion, with high inter-grader agreement. The study was performed using a fundus camera with a simple in-built infra-red pupillometer. Using this simple pupillometer we could compute the important pupillary dynamics parameters needed to evaluate ANS dysfunction. However, custom-built pupillometers used in some studies allows computation of a large number of parameters and should be noted as one of the limitations of our study^25^. The other limitation of our study is, being a diabetic screening camp based study, an equal number of patients could not be recruited in the study groups. However we believe that we have sufficient numbers in each group and use of the robust General Linear Model Repeated Measures analysis to overcome the influence of inter-eye dependency makes our deduction reasonably valid However, it would be interesting to verify our results in a large population based cohort sample, specially BPD and VPC parameters which although showed a decreasing trend, however was not found to be statistically significant.

Our study provides novel evidence of early ANS dysfunction in the natural history of diabetes, which progresses with retinopathy severity. Dynamic pupillometry is a valuable tool for the early detection of ANS dysfunction and its utility in DAN screening, which starts even in patients with no evidence of diabetic retinopathy, as observed in our study. This simple and inexpensive technique can potentially improve the outcome of patients with DAN by detecting it at sub-clinical stages. Exploring pupillary dynamics as a screening tool for DAN in larger population-based studies and its co-relation with the presence or absence of cardiovascular autonomic neuropathy is needed.
